# 

**Published:** 1876-02

**Authors:** 


Vol. XXXIII.

Number 2.

February, 1876.

NEW SERIES.
Vol. I.
Number 2.

### THE CHICAGO MEDICAL Journal and Examiner

PUBLISHED MONTHLY.

**EDITOR:**
WILLIAM H. BYFORD, A.M., M.D.

**ASSOCIATE EDITORS:**
JAMES H. ETHERIDGE, M.D. | JAS. NEVINS HYDE, A.M., M.D.
NORMAN BRIDGE, M.D. | FERD. C. HOTZ, M.D.

Published under the auspices of the
*CHICAGO MEDICAL PRESS ASSOCIATION.*

**TERMS—FOUR DOLLARS PER ANNUM, IN ADVANCE.**
**POSTAGE FREE.**

CHICAGO:
W. B. KEEN, COOKE & CO., Publishers,
Nos. 113 and 115 State Street.

